# Distinct Structural Features of G Protein-Coupled Receptor Kinase 5 (GRK5) Regulate Its Nuclear Localization and DNA-Binding Ability

**DOI:** 10.1371/journal.pone.0062508

**Published:** 2013-05-02

**Authors:** Laura R. Johnson, James D. Robinson, Katrina N. Lester, Julie A. Pitcher

**Affiliations:** Medical Research Council Laboratory for Molecular Cell Biology and Department of Neuroscience, Physiology and Pharmacology, University College London, London, United Kingdom; Rush University Medical Center, United States of America

## Abstract

G protein-coupled receptor kinases (GRKs) act to desensitize G protein-coupled receptors (GPCRs). In addition to this role at the plasma membrane, a nuclear function for GRK5, a member of the GRK4 subfamily of GRKs, has been reported. GRK5 phosphorylates and promotes the nuclear export of the histone deacetylase, HDAC5. Here we demonstrate that the possession of a nuclear localization sequence (NLS) is a common feature of GRK4 subfamily members (GRKs 4, 5 and 6). However, the location of the NLS and the ability of these GRKs to bind DNA *in vitro* are different. The NLSs of GRK5 and 6 bind DNA *in vitro*, whilst the NLS of GRK4 does not. Using mutants of GRK5 we identify the regions of GRK5 required for DNA-binding *in vitro* and nuclear localization in cells. The DNA-binding ability of GRK5 requires both the NLS and an N-terminal calmodulin (CaM)-binding site. A functional nuclear export sequence (NES), required for CaM-dependent nuclear export of the kinase, is also identified. Based on our observations we propose a model to explain how nuclear localization of GRK5 may be regulated. Notably, the nuclear localization of GRK5 and 6 is differentially regulated. These results suggest subfamily specific nuclear functions for the GRK4 subfamily members. Identification of GRK specific small molecule inhibitors of nuclear localization and/or function for the GRK4 subfamily may thus be an achievable goal.

## Introduction

The G protein-coupled receptor kinase (GRK) family of serine/threonine kinases were originally identified based on their ability to phosphorylate agonist occupied G protein-coupled receptors (GPCRs). GRK-mediated GPCR phosphorylation leads to recruitment of an arrestin family member, Arrestins 1–4, to the phosphorylated receptor. Arrestin binding sterically inhibits signaling via heterotrimeric G proteins, targets the receptor for internalization and initiates G protein-independent, arrestin-dependent, signal transduction [1]. Since their discovery the identification of numerous binding partners and non-GPCR substrates of the GRKs has revealed additional diverse functional roles for these enzymes, including in the nucleus [2], [3].

The seven members of the GRK family are grouped into three subfamilies; the GRK1 (GRK1 and 7), GRK2 (GRK2 and 3) and GRK4 (GRK4, 5 and 6) subfamilies [4]. We, and others, have shown that GRK5 and GRK6, members of the GRK4 subfamily, contain nuclear localization sequences (NLSs) [5], [6]. Indeed, GRK5 has been shown to contain a DNA-binding NLS and its nuclear localization is regulated by GPCR activation [5]. Nuclear localization of GRK5 is required for GRK5-mediated phosphorylation and subsequent nuclear export of the class II histone deacetylase (HDAC) HDAC5 [7]. In rat ventricular myocytes nuclear export of HDAC5 relieves HDAC5-mediated inhibition of transcription mediated by the transcription factor myocyte enhancer-2 (MEF2) and promotes MEF2-dependent expression of pro-hypertrophic genes [7]. Transverse aortic constriction (TAC) of mice with cardiac specific overexpression of GRK5 induces exaggerated, pathological hypertrophy as compared to wild type littermate controls [7]. The ability of GRK5 to inhibit HDAC5 activity is thought, at least in part, to underlie its ability to promote this response. The pathological hypertrophy observed in these mice includes impaired left ventricular function and higher mortality. This phenotype is not observed in mice with cardiac overexpression of a mutant form of the kinase in which the function of the NLS is ablated (GRK5ΔNLS) [7].

Notably, expressing the N-terminus of GRK5 (GRK5-NT) has been shown to lead to accumulation and stabilization of the IκBα/NF-kB complex in the nucleus and inhibition of NF-κB activity [8]. Intracardiac injection of an adenovirus encoding GRK5-NT reduces cardiac mass in spontaneously hypertensive rats and prevents the development of phenylephrine-induced left ventricular hypertrophy in Wistar Kyoto rats [9]. It remains to be determined to what extent the ability of GRK5 to bind IκBα in the context of the intact kinase [8], [10] is responsible for the exaggerated cardiac hypertrophy observed following TAC in transgenic mice with cardiac overexpression of GRK5.

GRK5-mediated HDAC5 phosphorylation and the ability of GRK5 to regulate IκBα function represent two mechanisms whereby cardiac overexpression of GRK5 could cause pathological cardiac hypertrophy in response to pressure overload in mice. However, the extent to which either of these mechanisms are actually responsible for GRK5-dependent cardiac hypertrophy *in vivo* and whether they represent the only means by which GRK5 promotes hypertrophy is not known. Similarly, we have very little information regarding how TAC promotes nuclear localization of GRK5 and whether the ability of GRK5 to bind DNA is required for GRK5-dependent hypertrophy. In this study we report the identification of an NES sequence in GRK5 and describe a variety of GRK5 mutants that differ in their ability to localize to the nucleus and/or their ability to bind DNA. These mutant constructs provide insights into the mechanisms regulating nuclear localization of GRK5 and provide useful tools for future dissection of the role of GRK5 *in vivo*.

GRK5 is upregulated in the left ventricle in dilated cardiomyopathy and left ventricular overload disease [11]. It is tempting to speculate, by analogy with the pressure overload model in transgenic mice, that elevated levels of GRK5 may be contributing to disease progression. A more detailed understanding of the regulatory mechanisms controlling the nuclear localization and functions of GRK5 may ultimately reveal novel druggable targets for the treatment of these disease states.

In this study we also demonstrate that possession of an NLS is a common feature of the GRK4 subfamily of GRKs. However, the location and DNA-binding properties of the NLS differs between subfamily members. Furthermore although binding to calmodulin (CaM) promotes nuclear export of GRK5, nuclear localization of GRK6A is unaffected by this treatment. Thus even though both kinases bind CaM, and have NLSs with similar locations and DNA-binding properties their nuclear export is differentially regulated. Although a nuclear function for GRK5 has been identified, a role for GRK4 and GRK6 in the nucleus remains to be elucidated. The observation that the nuclear export of GRK5 and GRK6A are differentially regulated and that GRK4 fails to bind DNA *in vitro* suggests subfamily specific nuclear functions for the GRK4 subfamily of GRKs.

To summarize, the aim of the experiments detailed in this study were, through analysis of mutant GRK5 constructs, to gain more information on the regulatory mechanisms controlling its nuclear localization. Nuclear functions for GRK5 are only just beginning to be characterized. Our study identifies a mutant of GRK5 that reveals that nuclear localization and the ability to bind DNA are dissociable properties of the kinase. This mutant will be a useful tool for probing the functional relevance of the DNA-binding ability of GRK5 *in vivo*. The identification of NLSs in other GRK4 subfamily members further indicates that investigating potential nuclear functions for these enzymes may also be a fruitful exercise.

## Materials and Methods

### Cell Culture

All cells were maintained in Dulbecco's modified Eagle medium (GIBCO) containing 10% fetal calf serum (Sigma) and penicillin and streptomycin (100 IU of penicillin and 100 mg of streptomycin/ml; Sigma) at 37°C, 5% CO2.

### Plasmids

**Figure 1:**
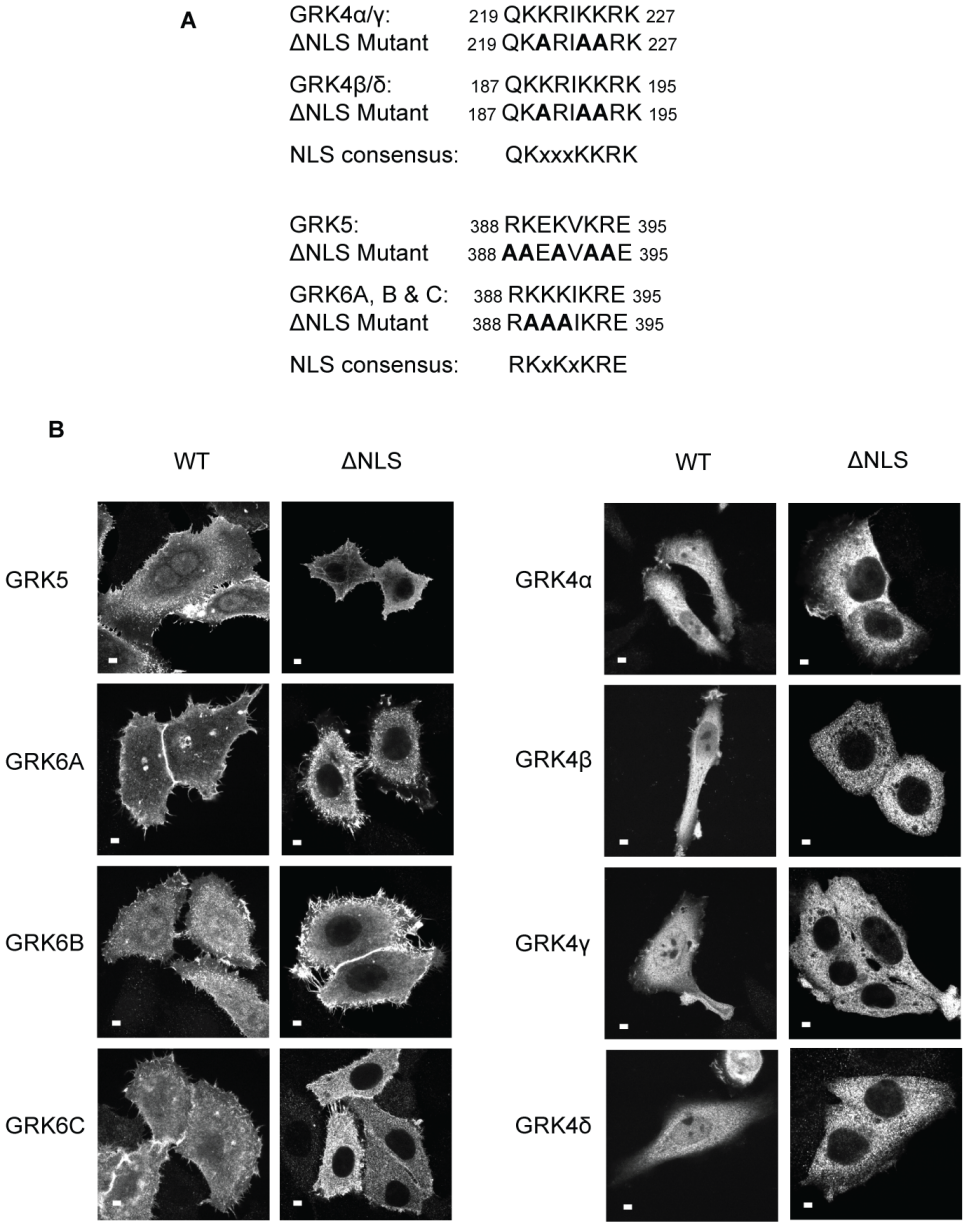
The GRK4 subfamily members contain a functional NLS. **A.** Regions of the catalytic domains of GRKs 4, 5 and 6 containing putative NLSs as predicted by PredictNLS [18]. The consensus NLS sequence and the position of amino acids mutated to alanine in GRKΔNLS mutants (indicated in bold) is also shown. **B.** GRK5, GRK6A, B and C, and GRK4a, b, g and d, together with their respective ΔNLS mutants were expressed in HEp2 cells and visualized by indirect immunofluorescence as described in Materials and Methods. Scale bars 10 mm.

The plasmids for pRK5-GRK5 [12], pRK5-GRK5ΔNLS [5], pRK5-GRK5NTPB [12], pRK5-GRK5CTPB [12], pRK5-GRK5AP [13], pRK5-GRK5K215R [12] and pcDNA3-GRK2 [14] have been described previously. pRK5-GRK5 and pcDNA3-GRK2 were used as a templates to create the GRK5ΔNES and GRK2ΔNES mutants respectively using the QuikChange site-directed mutagenesis kit (Stratagene). Hydrophobic residues between 259–265 (GRK5) and 267–264 (GRK2) were mutated to alanine residues (see Figure 1A) using the following primers: GRK5 sense primer, 5′ - GCC TAC GAG ACC AAG GAT GCC **GCG** TGT **GCA GCT GCT** ACC **GCC** ATG AAC GGC GGG GAC C – 3′; GRK5 anti-sense primer, 5′ – G GTC CCC GCC GTT CAT **GGC** GGT **AGC AG**′**C TGC** ACA CGC GGC ATC CTT GGT CTC GTA GGC – 3′. GRK2 sense primer, 5′ – CAC ACA CCG GAC AAG **GCC** AGC **GCA GCT GCT** GAT **GCC** ATG AAC GGC GGG GAC C – 3′; GRK2 anti-sense primer, 5′ – G GTC CCC GCC GTT CAT **GGC** ATC **AGC AG**′**C TGC** GCT **GGC** CTT GTC CGG TGT GTG – 3′. Nucleotides in bold encode the mutated amino acids.

pCMV5-GRK6A/B/C (human) were generous gifts from Dr Mario Tiberi (Ottawa Health Research Institute, Ottawa) [15]. pCMV5-GRK6A/B/C was used as a template to create GRK6ΔNLS mutants using the QuikChange site-directed mutagenesis kit (Stratagene). Lysine residues 389–391 were mutated to alanine residues using the following primers: sense primer, 5′ - CG CCC TTC CAG CAG AGG **GCA GCT GCT** ATA AAG CGC GAA GAG GTG GAG CGG C – 3′; anti-sense primer, 5′ – G CCG CTC CAC CTC TTC GCG CTT TAT **AGC AGC TGC** CCT CTG CTG GAA GGG CG – 3′. Nucleotides in bold encode the mutated amino acids.

pRK5-GRK4α/β/γ/δ (human) were generous gifts from Richard Premont, (Duke University, North Carolina) [16]. pRK5- GRK4α/β/γ/δ was used as a template to create GRK4ΔNLS mutants using the QuikChange site-directed mutagenesis kit (Stratagene). Three lysine residues in the predicted NLS of GRK4 splice variants were mutated to alanine residues using the following primers: sense primer, 5′ – GCC TGC AAA AAG CTA CAA AAA **GCT** AGA ATA **GCT GCT** AGG AAA GGT GAA GCT ATG GC– 3′; anti-sense primer, 5′ - GC CAT AGC TTC ACC TTT CCT **AGC AGC** TAT TCT **AGC** TTT TTG TAG CTT TTT GCA GGC – 3′. The location of the NLS in the splice variants of GRK4 is as follows; α and γ, residues 219–227, lysines 221, 224 and 225 mutated to alanine; β and δ, 187–195, lysine residues 189, 192 and 193 mutated to alanine. Integrity of mutant constructs was confirmed by DNA sequencing.

### Immunofluorescence

HEp2 cells (ATTC) were transfected by electroporation as described previously [5]. Briefly, cells treated with calcium ionophore were incubated with 25 µM A23187 (Calbiochem) for 15 min, unless otherwise indicated, in medium supplemented with 2 mM CaCl_2_ at 37°C, 5% CO2. Cells were subsequently immunolabelled with a mouse anti-GRK4-6 antibody (1∶300 dilution, Upstate) for 1 hr at room temperature. Confocal images were taken at room temperature using a Bio-Rad MRC 1024 laser scanning confocal system with Nikon Plan Apo 60x oil immersion lens and Optiphot 2 microscope using Bio-Rad Lasersharp 2000 software to acquire the images. The confocal plane shown represents a plane through the center of the nucleus as assessed by Hoechst staining. Images shown in a single figure were taken using the same microscope settings to ensure that cells with similar levels of GRK expression were compared within an experiment. Images were optimized for contrast in Adobe Photoshop, but no further manipulations were made. To quantify ionophore-dependent nuclear export at least 50 appropriately transfected cells were counted and GRK distribution scored as nuclear/cytosolic. Cells were deemed to show a cytosolic distribution if no transfected protein was detected in the nucleus. At least three separate transfections were scored per experiment.

### DNA binding assay

As described previously [5], briefly, equal amounts of GRK (as assessed by Western blotting, approximately 15 mg of protein) were incubated with 25 µl of native DNA-cellulose (Amersham) or cellulose (Sigmacell Cellulose type 50; Sigma) in a final volume of 100 µl of cold DNA binding buffer (10 mM HEPES [pH 7.4], 1 mM MgCl_2_, 0.1% Triton X-100, 3 mM dithiothreitol, 0.1 M NaCl, 0.05 mM EDTA) for 1 hr at 4°C. Following incubation, the resin was washed and the amount of GRK retained on the resin determined by Western blot analysis. Films were quantified using a densitometer (Bio-Rad). In each experiment a control and experimental value were compared and significance determined using a *t* test. For competition assays, purified GRK6A (150 ng) was preincubated with the stated amounts of DNA (sonicated, calf thymus; Amersham) or RNA (calf liver type IV; Sigma) for 30 min on ice before the DNA-binding assays were performed.

### GRK6A purification

GRK6A was expressed in, and purified from, baculovirus-infected SF9 cells as previously described [17].

## Results

### Nuclear localization is a common feature of the GRK4 subfamily of GRKs

The GRK4 subfamily contains three kinases, GRK4, GRK5 and GRK6. Four splice variants of human GRK4 (α, β, γ, δ) and three of GRK6 (A, B and C) have been identified. GRK5 contains an NLS located between amino acids 388 and 395 (^388^RKEKVKRE^395^) [5]. Mutation of basic amino acids in this sequence to alanine residues results in the nuclear exclusion of the mutant kinase (GRK5ΔNLS) ([5] and Figure 1A) without affecting its kinase activity [5]. The other members of the GRK4 subfamily, GRKs 4 and 6, are predicted to have putative NLSs (predicted by PredictNLS, an automated tool for the analysis and determination of NLS, Figure 1A and [18]) and are present in the nucleus of transfected cells (Figure 1B). We thus sought to determine if possession of a functional NLS is a feature common to all GRK4 subfamily members. Mutation of basic amino acids present in the predicted putative NLSs to alanine residues (Figure 1A) causes nuclear exclusion of all the splice variants of GRK4 and GRK6 (Figure 1B). These data confirm that all members of the GRK4 subfamily contain functional NLSs. Of note is the fact that the NLS of GRKs 5 and 6 is located in the same region of the kinase whereas the NLS of GRK4 is located more N-terminal to that of GRK5 and 6 (Figure 1A). Mutation of the GRK6A NLS also appears to disrupt its plasma membrane localization (Figure 1B). The mutation of three positively charged lysine residues may reduce the affinity of the kinase for membrane phospholipids, or alternatively may affect the positioning of the C-terminal amphipathic helix or the palmitoylation status of GRK6A [6]. The GRK2 subfamily, that is GRK2 and GRK3, are not predicted to contain NLSs [18].

### GRK5 and 6, but not 4, binds DNA *in vitro*

**Figure 2:**
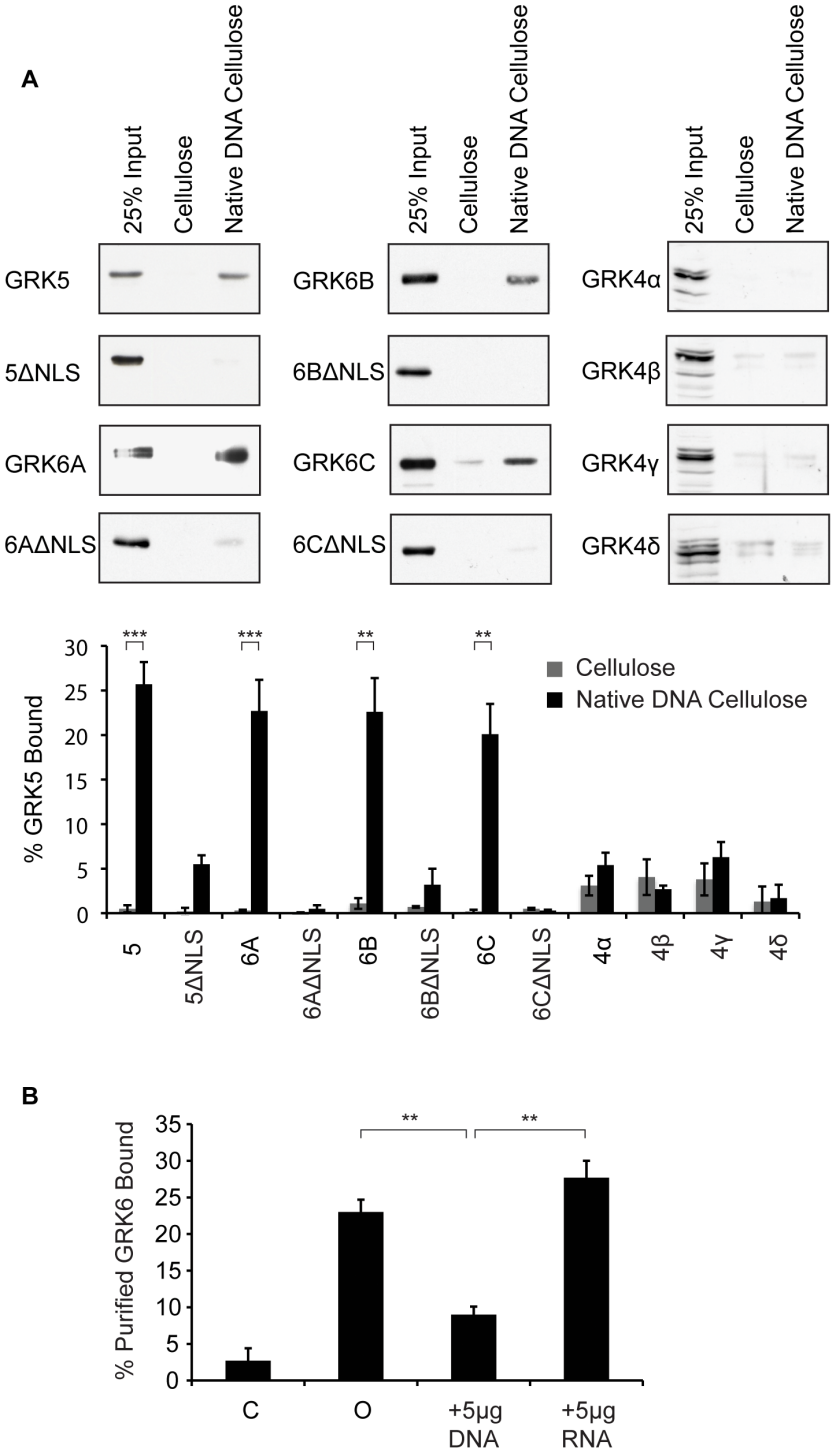
GRKs 5 and 6 bind DNA in vitro. **A.** Cos-7 cell lysates (∼15 µg) expressing approximately equivalent amounts of GRK5, GRK6A, B and C and ΔNLS mutants thereof or GRK4a, b, g and d were incubated with native DNA-cellulose or cellulose, after extensive washing the amount of bound GRK was determined by Western blot analysis. Blots were quantified as described in Materials and Methods. Representative blots are shown along with quantification representing mean values ± standard errors of the mean for at least three separate determinations. **, P<0.01; ***, P<0.001. **B.** Purified GRK6A (150 ng) was incubated with native DNA-cellulose or the cellulose support (C) following a 30 min incubation without (0) or with the indicated amounts of DNA or RNA. The amount of GRK6A retained by the resin was determined as described in A. The data shown represent the mean values ± standard errors of the mean five separate determinations. **, P<0.01.

GRK5 binds DNA *in vitro* and DNA-binding is mediated, at least in part, by its NLS ([5] and Figure 2A). To determine if the other GRK4 subfamily members contain DNA-binding NLSs Cos-7 cell lysates, expressing wildtype or ΔNLS mutant kinases, were incubated with native DNA-cellulose and the amount of GRK bound to the resin determined. For each GRK construct ∼15 µg of cell lysate protein containing equivalent amounts of GRK, as assessed by Western blot, were used in each binding assay. As shown in Figure 2A all the GRK6 splice variants bind to DNA-cellulose *in vitro*. When expressed as percent of the total input bound 22.7 ± 3.5%, 22.6 ± 3.8%, and 20.1 ± 3.4% of, respectively, GRK6A, B and C bound to DNA cellulose, that this represents specific binding to DNA is indicated by the observation that GRK6 does not bind the cellulose support (for GRK6A 0.3 ± 0.1%; GRK6B 1.1 ± 0.6% and GRK6C 0.2 ± 0.2% of the load bound) (Figure 2A). GRK6A, B and C ΔNLS mutants also fail to bind DNA, with respectively, 0.5 ± 0.4%, 3.2 ± 1.8% and 0.3 ± 0.1% of the mutant kinases binding to the resin (Figure 2A), suggesting that the NLS of GRK6 is part of its DNA binding site. GRK6A, B and C are thus similar to GRK5 in that they bind specifically to DNA via, at least in part, a DNA-binding NLS. In marked contrast, none of the splice variants of GRK4 bind DNA (5.6 ± 1.4%, 2.7 ± 0.4%, 6.3 ± 1.7% and 1.7 ± 1.5% of the GRK4 α, β, γ and δ splice variants bound to the resin) (Figure 2A). The amount of GRK4 bound to DNA-cellulose was not significantly different from that bound by the cellulose support. The observation that GRKs 5 and 6, but not 4, bind DNA *in vitro* suggests distinct nuclear functions for these enzymes.

GRK5 binds DNA directly [5], to determine if GRK6 binding to DNA is direct and specific we repeated the *in vitro* DNA binding assay using purified GRK6A. Approximately 23 ± 1.7% of the purified GRK6A (150 ng) incubated with native DNA-cellulose was retained by the resin (Figure 2B). Preincubation of the purified kinase with DNA, but not RNA, inhibited binding to DNA-cellulose (Figure 2B) suggesting that indeed, like GRK5, GRK6 binds directly to DNA. That RNA fails to displace DNA-bound GRK6A suggests that binding to DNA is specific.

### GRK5 contains a nuclear export sequence (NES)

**Figure 3:**
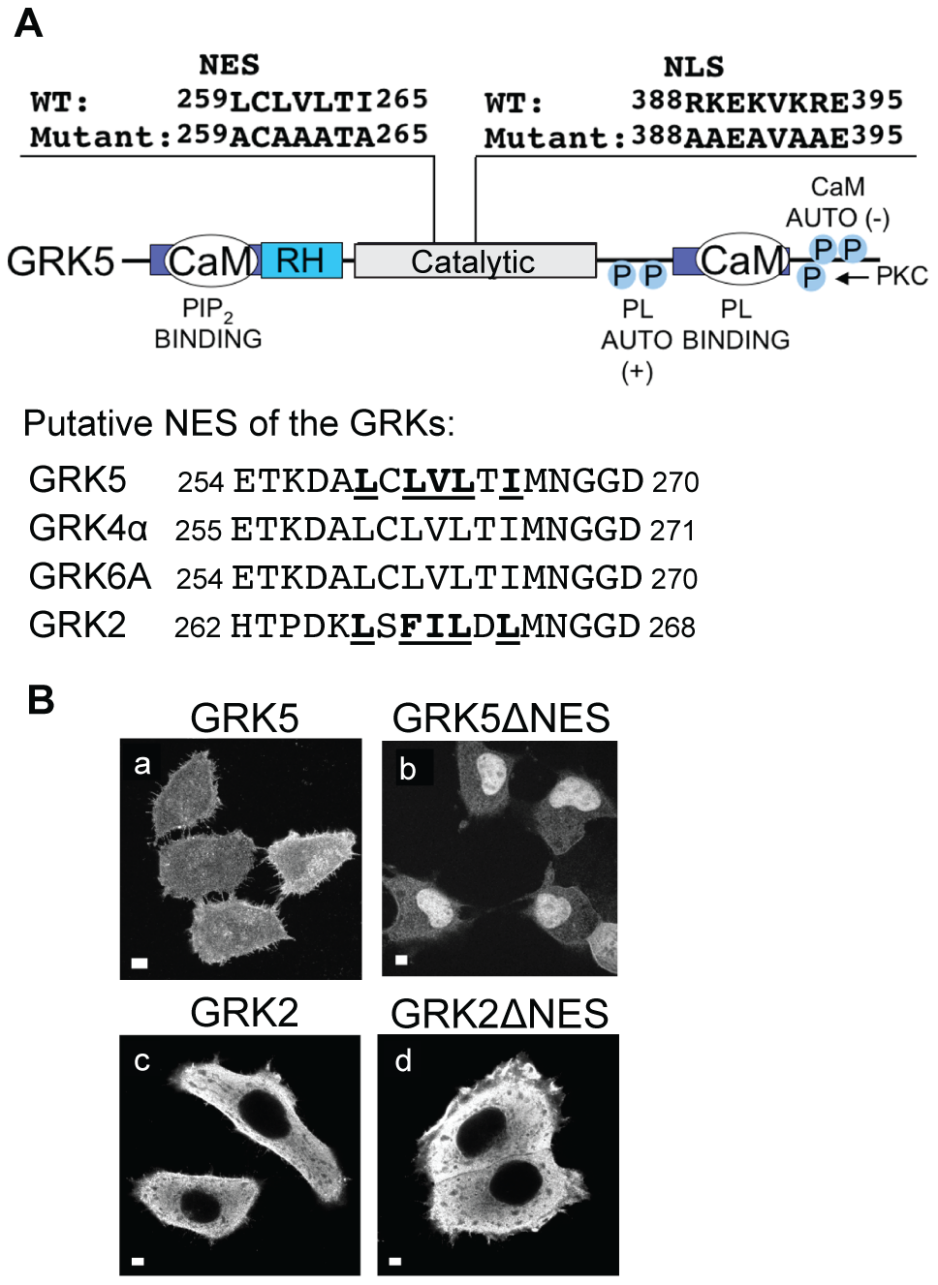
GRK5 contains a nuclear export sequence. **A.** Domain structure of GRK5 indicating location and sequence of the NLS and putative NES. Sequence alignment of the GRK4 subfamily members showing putative NESs together with the homologous sequence in GRK2. Residues mutated in ΔNLS mutants are indicated in bold. Numbers indicate amino acid sequence location. CaM, calmodulin-binding domain; RH, regulator of G-protein signaling (RGS) homology domain; PL, phospholipid; P, phosphorylation site; Auto, autophosphorylation; PKC; protein kinase C. **B.** Wildtype GRK5 (a), GRK5ΔNES (b), GRK2 (c) GRK2ΔNES (d) were overexpressed in HEp2 cells and their cellular distribution assessed by indirect immunofluorescence. Scale bars, 10 mm.

GRK5 can function in the nucleus to regulate cardiac hypertrophy [7]. The identification of functional NLSs in the other GRK4 subfamily members suggests that they too have nuclear activities. Since GRKs 5 and 6, but not GRK4, bind DNA *in vitro* it seems likely that the GRK4 subfamily of GRKs may have distinct nuclear activities. GRK5 expression *in vivo* promotes TAC-induced activation of a program of gene transcription required for cardiac hypertrophy [7]. A detailed understanding of how GRK5 mediates this effect requires identification of the specific properties of the kinase responsible for regulating its nuclear localization and functions. This information may also, by analogy, highlight common themes and differences between GRKs 4, 5 and 6. We thus sought to examine whether, in addition to its NLS, GRK5 contains other motifs responsible for regulating its nuclear localization. A putative NES was identified in GRK5 using the NESbase 1.0 search program [19]. The predicted NES includes residues 259–265 of GRK5 and is located within the catalytic domain of the enzyme 129 amino acids N-terminal to the NLS (Figure 3A). Mutation of hydrophobic residues in this sequence to alanine (Figure 3A, residues shown in bold), resulted in a mutant form of GRK5 (GRK5ΔNES) that is predominantly confined to the nucleus when expressed in HEp2 cells, in contrast to wildtype GRK5 that is distributed throughout the nucleus and cytoplasm (Figure 3B, panels a and b). These observations confirm that GRK5 contains a functional NES. Notably, the GRK5ΔNES mutant phosphorylates HDAC5 when overexpressed in cardiomyocytes [7], demonstrating that it remains catalytically active. The sequence encoding the NES of GRK5 is conserved in all GRK4 subfamily members, GRKs 4, 5 and 6 (Figure 3A) suggesting that the possession of an NLS and an NES may be a common feature of this subfamily of GRKs. NESbase 1.0 [19] failed to identify a consensus NES sequence in GRK2 or 3, members of the GRK2 subfamily of GRKs. Consistent with this observation, mutation of hydrophobic residues in GRK2 corresponding to the NES of GRK5 (Figure 3A, residues in bold) had no effect on the cytosolic distribution of this kinase (Figure 3B, panels c and d). To date we have failed to identify an NES in GRK2. These results suggest that possession of an NLS and an NES may distinguish GRK4 from GRK2 subfamily members.

### Calcium-dependent nuclear export of GRK5 is NES-dependent

**Figure 4:**
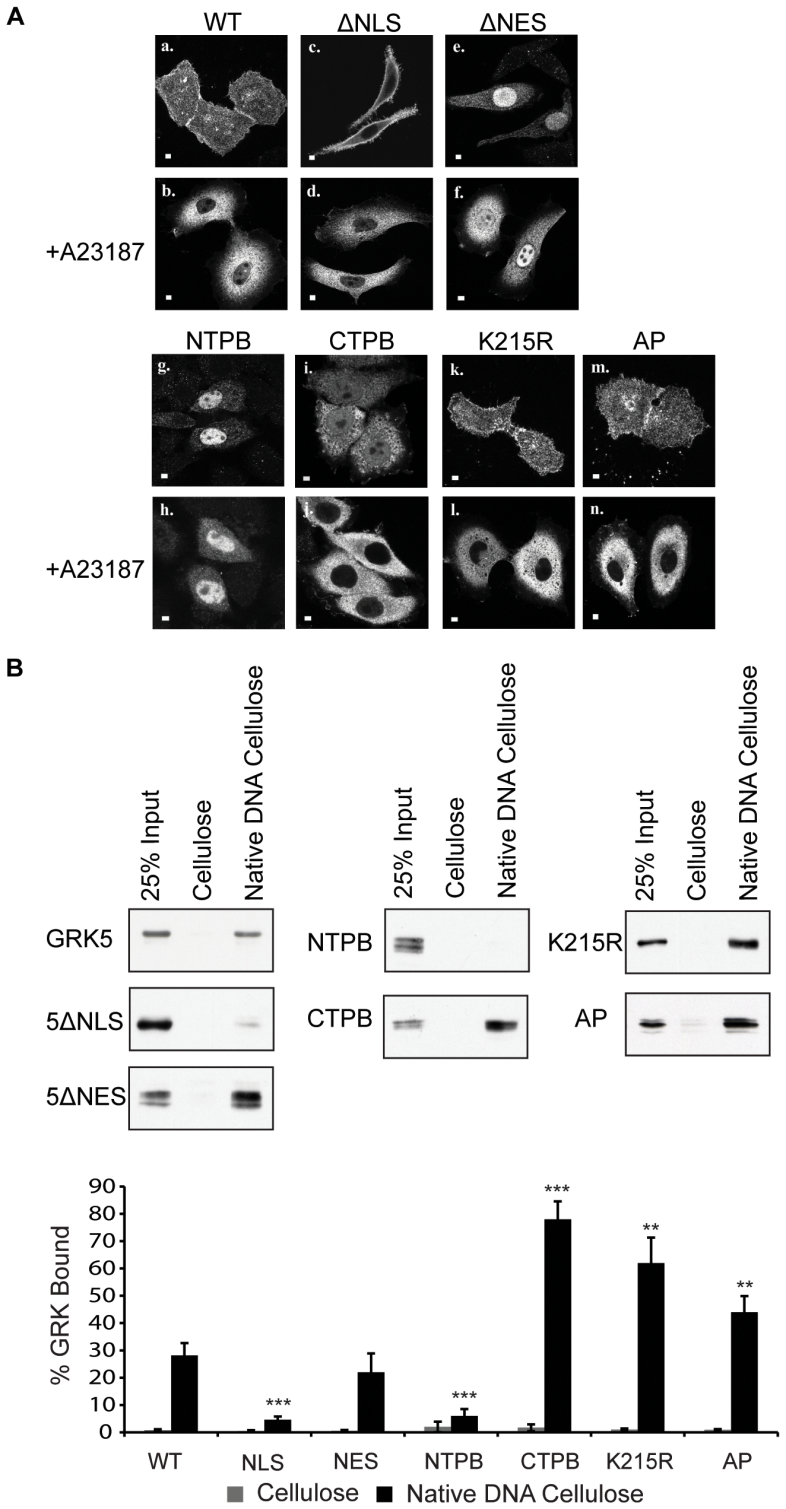
Structural determinants of GRK5 required for nuclear localization and DNA binding in vitro. **A.** Confocal microscopy showing cellular distribution of GRK5, and mutants thereof, overexpressed in HEp2 cells +/− A23187 (25 mM, 15 min) treatment. WT, wildtype GRK5; ΔNLS, GRK5ΔNLS; ΔNES, GRK5ΔNES; NTPB, N-terminal CaM binding site mutant; CTPB, C-terminal CaM binding site mutant; K215R, catalytically inactive GRK5; AP, autophosphorylation site mutant. Scale bars are 10 mm. **B.** DNA-binding assays, described in Materials and Methods, were performed on Cos-7 cell lysates overexpressing approximately equal amounts of GRK5, or the mutant GRK5 constructs indicated. Representative blots are shown along with quantification representing mean values ± standard errors of the mean for six separate determinations. Statistical significance is determined relative to wild type GRK5. **, P<0.01; ***, P<0.001.

The identification of both an NLS and NES in GRK5 suggests that its nuclear localization may be regulated. As shown previously, treatment with a calcium ionophore (A23187) promotes nuclear export of GRK5 ([5] and Figure 4A panels a and b). GRK5ΔNLS is excluded from the nucleus under basal conditions and thus remains cytoplasmic following treatment (Figure 4A, panels c and d). The nuclear localization of GRK5ΔNES is relatively unchanged following A23187 treatment, demonstrating that calcium-dependent nuclear export of GRK5 does indeed require its NES (Figure 4A, panels e and f). GRK5 contains two CaM-binding sites, one in its N-terminus (between residues 20–39) and one in its C-terminus (between residues 540–578) [4]. Previous work has demonstrated that binding of CaM to the N-terminal binding site of GRK5 is required for Ca^2+^/CaM-dependent nuclear export [5]. A mutant of GRK5 in which 5 basic amino acids in the N-terminal calmodulin (CaM)-binding site of GRK5 are mutated to alanine (GRK5NTPB) is nuclear in both the presence and absence of ionophore ([5] and Figure 4, panels g and h). In contrast, a mutant GRK5 construct lacking the C-terminal CaM binding site (GRK5CTPB) behaves as the wild type enzyme ([5] and Figure 4, panels i and j). The observation that both GRK5ΔNES and GRK5NTPB display similar nuclear localization, and are refractory to the effects of A23187 treatment, suggests that binding of CaM to the N-terminal binding site of GRK5 is responsible for altering the conformation of the kinase such that its NLS is hidden and NES exposed.

Analysis of these and other GRK5 mutants, previously uncharacterized with respect to their nuclear localization, reveals a panel of mutant kinase constructs that vary in their subcellular localization and nuclear export characteristics. As shown in Figure 4A wild type GRK5 (panels a and b), GRK5CTPB (panels i and j), GRK5K215R (a catalytically inactive mutant) (panels k and l), and GRK5AP (a mutant of GRK5 lacking autophosphorylation sites) (panels m and n) are uniformly distributed throughout the cytoplasm and nucleus and are exported in a CaM-dependent fashion. In contrast, GRK5ΔNES (panels e and f) and GRK5NTPB (panels g and h) are localized to the nucleus whilst GRK5ΔNLS (panels c and d) is cytoplasmic. The subcellular distribution of GRK5ΔNES, GRK5NTPB and GRK5ΔNLS are unaffected by ionophore treatment.

### The structural determinants of GRK5 required for DNA-binding

GRK5 binds DNA *in vitro* and this requires an intact NLS [5]. To examine if other structural features regulate DNA binding, the panel of GRK5 mutants shown in Figure 4A, were assessed for their ability to bind DNA *in vitro*. Consistent with previously reported findings wildtype GRK5 bound DNA-cellulose (28.2 ± 4.5% of the load bound) but not the cellulose support (0.8 ± 0.3% bound, Figure 4B) [5]. GRK5ΔNLS failed to bind DNA (4.6 ± 1.2% bound), confirming that an intact NLS is required for this interaction [5]. Analysis of the DNA-binding properties of the previously uncharacterized mutants reveals that the DNA-binding ability of GRK5ΔNES (22 ± 6.9% bound) is identical to that of the wild type enzyme (Figure 4B, NES) while that of GRK5NTPB (6 ± 2.5% bound) (Figure 2B, NTPB) is similar to that of GRK5ΔNLS. Thus despite having a similar nuclear localization GRK5ΔNES and GRK5NTPB exhibit very different DNA-binding abilities. The inability of GRK5NTPB to bind DNA suggests that, like the NLS, this region of the kinase may be in direct contact with DNA. Presumably these two regions form part, or all, of the DNA binding domain of GRK5.

GRK5CTPB, GRK5K215R and GRK5AP bind DNA more avidly than their wild type counterpart, respectively, 78 ± 6.6%, 62 ± 9.3% and 44 ± 5.9% of the load bound as compared to 28.2 ± 4.5% for the wild type enzyme (Figure 4B). These results suggest that binding of CaM to the C-terminal domain of the kinase, GRK5 catalytic activity, and GRK5 autophosphorylation negatively regulate DNA-binding. The binding of CaM to the C-terminal CaM binding site of GRK5 stimulates autophosphorylation at sites distinct from the ‘classical’ phospholipid-stimulated autophosphorylation sites (S484 and T485) [4]. Although the sites phosphorylated in the presence of CaM remain to be definitively mapped they reside in the C-terminus of the kinase between residues 579 and 585, i.e. at a site more C-terminal than phospholipid stimulated autophosphorylation sites [4]. Taken together these results suggest that the addition of negatively charged phosphate groups to serine/threonine residues in the C-terminus of GRK5 reduces its affinity for DNA. Preventing autophosphorylation by inhibiting kinase activity (GRK5K215R), removing the sites of autophosphorylation (GRK5AP) or by mutating the C-terminal CaM-binding site (GRK5CTPB) would thus enhance DNA-binding. That GRK5CTPB binds most avidly to DNA (Figure 4B) may reflect an additional direct effect of CaM binding at this C-terminal binding site to reduce DNA-binding affinity.

### The nuclear export of GRK6A is not Ca^2+^-dependent

**Figure 5:**
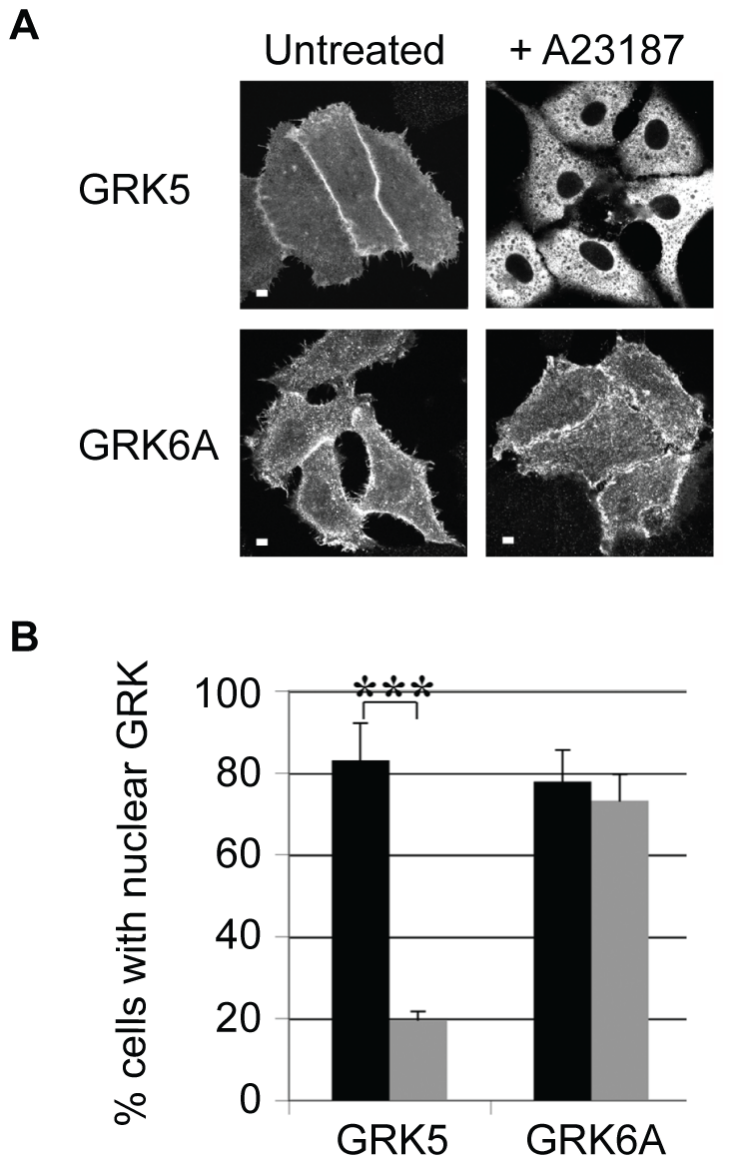
Differential regulation of the nuclear localization of GRK5 and GRK6A. **A.** Confocal microscopy showing cellular distribution of GRK5 and GRK6A overexpressed in HEp2 cells +/− A23187 (25 µM, 15 min) treatment. Scale bars are 10 mm. **B.** Quantification of experiments performed as described in A. Black bars represent untreated and grey bars A23187 treated cells. Nuclear localization of the GRK construct was scored as described in Materials and Methods. The data shown represent the mean values ± standard errors of the mean for at least three separate transfections, at least 50 cells were scored per transfection. ***, P<0.001.

Since both GRK5 and GRK6 contain NLSs of similar sequence, location and DNA-binding properties we examined whether the nuclear export of these kinases was similarly regulated. As shown in Figure 4, Figure 5 and [5], treatment with the calcium ionophore A23187 promotes nuclear export of GRK5 in a manner that is dependent on CaM-binding to an N-terminal polybasic region. In marked contrast the nuclear localization of GRK6A, which is also a CaM-binding protein, is unaffected by ionophore treatment (Figure 5, A and B). Thus despite similarities in their NLSs and N-terminal CaM-binding domains these kinases exhibit marked differences in the regulatory mechanisms controlling their nuclear localization. The signals, if any, responsible for promoting nuclear export of GRK6A, remain to be determined.

## Discussion

All members of the GRK4 subfamily contain functional NLSs, however, the NLS sequence of GRKs 5 and 6 differ from that of GRK4 in sequence, location and DNA-binding properties. Thus the NLS of GRKs 5 and 6, but not 4, bind DNA *in vitro*. This data suggests nuclear function(s) for GRK4 distinct from that of GRKs 5 and 6. The NLS of GRK4 is similar in sequence and location to predicted putative NLSs in GRK1 and GRK7 [18] rather than the DNA-binding NLSs of GRKs 5 and 6 [18]. The GRK2 subfamily does not contain putative NLSs, as assessed by the PredictNLS search engine, suggesting that these kinases may be the only GRK family members without nuclear functions.

**Figure 6:**
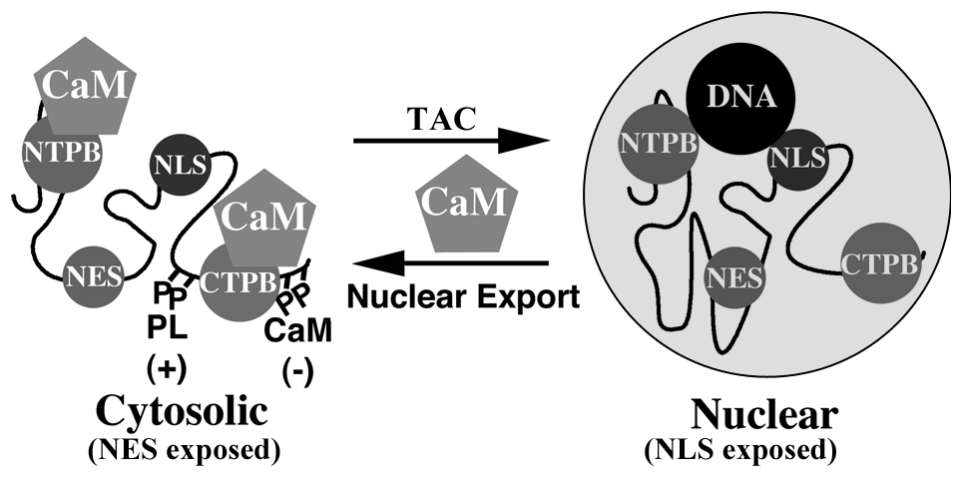
Model illustrating potential mechanisms regulating DNA-binding and nuclear export of GRK5. GRK5 resides in the nucleus, free of CaM, in a conformation in which its NLS is exposed and NES hidden. DNA-binding is mediated, at least in part by its NLS and N-terminal CaM binding domain (NTPB). CaM binding to the N-terminus of GRK5 exposes the NES of GRK5 facilitating its nuclear export. CaM-binding to the C-terminal CaM-binding site of GRK5 and autophosphorylation of the kinase at multiple sites within its C-terminus, although not directly promoting nuclear export, facilitates dissociation of GRK5 from DNA. Transverse aortic constriction (TAC) has been shown to promote nuclear accumulation of GRK5 *in vivo* [7].

Analysis of the subcellular localization and DNA-binding properties of the seven GRK5 mutants used in this study suggests a potential model whereby GRK5 nuclear localization may be regulated (Figure 6). The wild type kinase is distributed throughout the cytoplasm and nucleus of transfected cells suggesting two populations of GRK5 in which, respectively, the NES or NLS are exposed. GRK5ΔNLS is cytosolic and GRK5ΔNES nuclear, suggesting an obligate requirement for these two motifs in establishing, respectively, a nuclear or cytosolic location. Endogenous Ca^2+^/CaM appears to be responsible for exposing the NES in the cytosolic pool of transfected wild type GRK5 since GRK5NTPB, which lacks the N-terminal CaM binding site, is almost entirely nuclear. The observation that both GRK5ΔNES and GRK5NTPB are not exported from the nucleus in a Ca^2+^/CaM-dependent fashion further supports this contention since it demonstrates that CaM-dependent nuclear export is both NES and CaM-dependent. A role for CaM in mediating nuclear export has also been proposed for Ca^2+^/CaM dependent protein kinase I-α where CaM-binding promotes binding of the kinase to the CRM1 nuclear export complex [20].

In our model the nuclear pool of wild type GRK5 is postulated to be free of CaM (Figure 6). In the nucleus GRK5 may be bound to DNA; DNA binding requires both an intact NLS and N-terminal CaM binding site since mutation of either of these domains prevents DNA-binding *in vitro* (Figure 4B). Binding of CaM to the N-terminal CaM binding site of GRK5 may thus serve not only to expose the NES of GRK5, but may also contribute directly to its dissociation from DNA. GRK5K215, GRK5AP and GRK5CTPB all exhibit an enhanced DNA-binding ability *in vitro* as compared to the wild type enzyme (Figure 4B). This suggests that phosphate addition, and potentially CaM binding, to the C-terminus of the kinase contributes to reduce its affinity for DNA. These modifications do not, however, result in NES exposure since these mutant forms of the enzyme show a subcellular distribution identical to that of wild type GRK5 (Figure 4A). Overall our studies suggest a complex mechanism for regulating the conformation of GRK5 via interaction with regulatory proteins (CaM) or by post-translational modifications (autophosphorylation) (see Figure 6). Altering conformation is postulated to modulate NLS/NES exposure thereby affecting nuclear localization and DNA-binding properties of GRK5. Atypical protein kinase C-λ (αPKCλ) [21], and MAPKAP kinase 2 [22] also contain both an NLS and NES, however, for these enzymes phosphorylation-dependent activation, rather than CaM-binding, regulates NLS/NES exposure and nuclear localization. Active αPKCλ enters, whilst active MAPKAP kinase 2 exits, the nucleus [21], [22].

Despite having NLSs with similar locations and DNA-binding properties and both being CaM-binding proteins, the nuclear export of GRK5 is Ca^2+^-dependent whereas GRK6A appears to be refractory to this regulatory mechanism. CaM activation fails to promote nuclear export of GRK6A even though GRK6 binds CaM as evidenced by CaM-dependent inhibition of GRK6A-mediated rhodopsin phosphorylation [23]. It would thus be of interest to investigate the features of GRK6A responsible for conferring insensitivity to Ca^2+^-dependent nuclear export. Previous work has suggested that palmitoylation of GRK6A may be responsible for maintaining a cytosolic (plasma membrane localized) distribution of the kinase and that when the palmitoylation sites on GRK6A are mutated nuclear GRK6A can be detected [6]. These observations are consistent with a model in which regulating the palmitoylation status of GRK6A may regulate its nuclear localization. Notably in the context of the palmitoylation mutant of GRK6A, Jiang and colleagues demonstrated that mutation of amino acid K219 to Q leads to its nuclear exclusion in HEK cells [6]. This observation suggests that in this mutant background the basic amino acids located in an analogous position to the NLS of GRK4 may represent a functional NLS in GRK6. In support of this observation the mouse GRK6 splice variant GRK6D, which lacks the NLS identified in this study, but not that identified in [6], is located in the nucleus of Cos cells [24]. In this study we have identified a functional NLS (residues 388–395), which when mutated in the wild type kinase, causes nuclear exclusion of GRK6 in cells and prevents DNA-binding *in vitro*. Given the complexity of regulatory mechanisms controlling the nuclear localization of GRK5 it is not beyond the realms of possibility that both sequences may participate in regulating the nuclear localization of GRK6, potentially, in a cell type specific fashion. That the nuclear export of GRK5 and 6 is differentially regulated may suggest a common nuclear function that is differentially modulated following activation of specific receptor subsets, or alternatively, that GRK5 and 6 perform distinct nuclear functions. Additionally, it suggests that it may be possible to design subfamily specific small molecule regulators of the nuclear functions of GRK5 and 6A by selectively blocking their nuclear import/export.

The crystal structure of GRK6A has been solved and suggests that this kinase may exist as a dimer [25]. This is in contrast to the crystal structure elucidated for GRK2 that suggests a monomeric structure [26]. Mutations of GRK6A predicted to inhibit dimerization had no effect on GRK6A-mediated phosphorylation of rhodopsin *in vitro* [25]. It is thus possible that dimerization is required for other, perhaps nuclear, functions of GRK6A. DNA-binding of many transcription factors/co-regulators [27], [28] is dimerization-dependent. It would thus be of interest to examine whether the dimerization of GRK6A is required for DNA-binding. In the GRK6 dimer the two NLSs and the NTPB domains, which by analogy with GRK5 are likely to both be required for DNA-binding are located, respectively, at opposite ends of the dimer (between the αF-αG loop of the kinase [25]) and close to the dimer interface. It is thus tempting to speculate that the kinase dimer may wrap itself around DNA. Determination of the crystal structures of GRK5 and GRK4 that, respectively, do and don't bind DNA will elucidate whether the ability to dimerize is a feature specific to the DNA-binding GRKs (GRKs 5 and 6) or is a common feature of the GRK4 subfamily of GRKs.

This study highlights both a structural diversity between the GRK2 and GRK4 subfamilies of GRKs and a potential functional diversity within the GRK4 subfamily. A nuclear function for GRK5, phosphorylation and inhibition of the MEF2 repressor HDAC5, has been identified. Presumably GRK6 and GRK4 also have nuclear functions. What these are and whether they vary in a subtype, or splice variant, specific fashion remains to be determined. In this study we identify a functional NES in GRK5 and describe a number of mutants of this kinase that vary in their ability to localize to the nucleus or to bind DNA. Analysis of the hypertrophic response following TAC in transgenic mice with cardiac specific overexpression of these mutants is anticipated to reveal more information on the role of GRK5 in this *in vivo* model of pressure overload disease. Specifically the role GRK5 kinase activity, nuclear localization and DNA-binding ability plays in promoting pathological hypertrophy in response to TAC may reveal additional nuclear functions for this kinase. A more detailed understanding of the molecular features of GRK5 required to promote pathological hypertrophy may lead to the identification of regions on GRK5 or target molecules amenable to regulation via small molecule inhibitors.
